# Education and risk of incident dementia during the premotor and motor phases of essential tremor (NEDICES)

**DOI:** 10.1097/MD.0000000000004607

**Published:** 2016-08-19

**Authors:** Julián Benito-León, Israel Contador, Elan D. Louis, Stephanie Cosentino, Félix Bermejo-Pareja

**Affiliations:** a Department of Neurology, University Hospital “12 de Octubre”, Madrid, Spain; b Centro de Investigación Biomédica en Red sobre Enfermedades Neurodegenerativas (CIBERNED), Spain; c Department of Medicine, Complutense University, Madrid, Spain; d Department of Basic Psychology, Psychobiology and Methodology of Behavioral Science, University of Salamanca, Salamanca, Spain; e Department of Neurology, Yale School of Medicine; f Department of Chronic Disease Epidemiology, Yale School of Public Health; g Center for Neuroepidemiology and Clinical Neurological Research, Yale School of Medicine and Yale School of Public Health, New Haven, CT; h Cognitive Neuroscience Division of the Gertrude H. Sergievsky Center; i Taub Institute for Research on Alzheimer's Disease and the Aging Brain; j Department of Neurology, Columbia University Medical Center, New York, NY.

**Keywords:** cognitive reserve, dementia, education, essential tremor, population-based study, premotor symptoms

## Abstract

Individuals with late-onset essential tremor (ET) (e.g., older adults) seem to have an increased prevalence of mild cognitive impairment and dementia, and a higher risk of incident dementia. It is well-known that education has a protective role against dementia in individuals without a pre-existing neurologic disorder, but evidence regarding the maintenance of this effect during the premotor and motor phases of ET is unknown. Our aim was to determine the influence of education on the risk of dementia in a population-based cohort of ET patients and controls. In a prospective study (Neurological Disorders in Central Spain), participants ≥65 years old were evaluated twice: at baseline (1994–1995) and at follow-up (1997–1998). There were 3 groups: premotor (i.e., participants first diagnosed with incident ET at follow-up), prevalent ET (i.e., participants diagnosed with ET at baseline and at follow-up), and controls. Participants were stratified into lower education (≤primary studies) versus higher education (≥secondary studies) categories. Dementia risk was estimated using Cox proportional-hazards models (higher education control group = reference category). Among the participants, 3878 had a mean duration of follow-up of 3.2 years. Eight (16.7%) of 48 lower education premotor ET patients developed incident dementia versus 1 (3.3%) of 30 higher education premotor ET patients, 9 (7.1%) of 126 lower education prevalent ET patients, 7 (8.8%) of 80 higher education prevalent ET patients, and 92 (4.9%) of 1892 lower education controls (*P* < 0.001). In comparison to the higher education controls, the adjusted hazard ratios for incident dementia were 5.84 (lower education premotor ET, *P* < 0.001); 1.36 (higher education premotor ET, *P* = 0.76); 2.13 (lower education prevalent ET, *P* = 0.04); 2.79 (higher education prevalent ET, *P* = 0.01); and 1.66 (lower education controls, *P* = 0.01). Our results suggest that a higher educational attainment may ameliorate the risk of incident dementia during the premotor phase of ET, but not in the motor phase.

## 1 Introduction

Higher education is associated with a decreased risk of incident dementia^[1,2]^ and better maintenance of cognitive function in the setting of brain pathology.^[3,4]^ Functional imaging studies in aged populations have demonstrated that brains of people with higher education are more efficient in terms of functional connectivity.^[5]^ Indeed, education may contribute to the brain's capacity (e.g., synaptic density) to tolerate neuropathology (passive approach), and also an indicator of the brain's ability to compensate for damage using existing or alternative networks according to the active approach proposed by the cognitive reserve theory.^[6]^ Epidemiological studies suggest that through promoting these forms of reserve, education reduces the risk of developing dementia.^[4,7]^ Specifically, high reserve may influence the capability of individuals to cope with Alzheimer disease (AD) pathology for a longer period of time. However, the beneficial effect of education is not unlimited. At a certain point in the disease (i.e., “inflection point”), higher education seems to be associated with more rapid cognitive decline, suggesting that the burden of neuropathology has reached a level at which compensatory mechanisms begin to fail.^[6]^

Essential tremor (ET) is a progressive, aging-associated condition, characterized by cell loss (reduction in Purkinje cell number in some studies) and other types of changes (Lewy body formation) that traditionally occur in neurodegenerative disorders.^[8]^ Mild cognitive deficits, mainly in attention and frontal executive functions, verbal memory, and visuospatial processes have been reported in ET, which may be explained by frontal cortical or frontal cortical–cerebellar pathways dysfunction.^[9–13]^ Furthermore, cognitive deficits in ET might be not static and seem to progress at a faster rate than in normal older adults.^[12]^ In particular, individuals with late-onset ET (e.g., older adults) seem to have an increased prevalence of mild cognitive impairment and dementia,^[14,15]^ and a higher risk of incident dementia^[16]^ than those with earlier-onset ET.

Intriguingly, there is growing evidence that nonmotor symptoms may be an integral part of the clinical spectrum of ET^[17]^ and may even antedate tremor onset (e.g. depressive symptoms^[18]^ or faster cognitive decline).^[13]^ The existence of a stage of ET where affected subjects may be asymptomatic (“preclinical ET”) or where they may present with a variety of nonmotor symptoms and/or subtle motor signs that do not meet current diagnostic criteria (“prodromal ET”) may be denominated premotor ET. However, studies have yet to prospectively assess whether individuals with premotor ET have an increased risk of incident dementia or cognitive decline. Moreover, it is unclear as to whether factors that have been shown to ameliorate dementia risk in other neurodegenerative diseases influence dementia risk in ET and premotor ET.

It is well-known that education has a protective role against dementia in individuals without a pre-existing neurologic disorder,^[7]^ but evidence regarding the maintenance of this effect in people suffering from ongoing neurological disorders is very scarce. In Parkinson disease (PD), education was shown not to influence progression of cognitive impairment^[19]^ or conversion to dementia.^[20]^ Whether education influences the risk of dementia among patients in the premotor phase of PD is unknown.^[21]^ Interestingly, a composite measure of cognitive reserve was associated with slower rate of cognitive change and slower rate of brain atrophy in 2 brain structures (putamen, caudate) in prodromal Huntington disease.^[22]^ Therefore, it is plausible that education may ameliorate the risk of dementia in the premotor phase of ET, but not in prevalent ET patients where neuropathology and/or abnormal brain connectivity is more severe as compared with premotor phases.^[23]^

We examined the influence of education on risk of incident dementia in a population-based cohort of premotor ET patients (participants diagnosed with incident ET at follow-up, but not at baseline), prevalent ET patients (participants diagnosed with ET at baseline), and controls (participants not diagnosed with ET at baseline or follow-up). In line with the previously reported finding that later-onset ET is more likely to be associated with dementia than early-onset ET, we hypothesized that risk of dementia is increased in premotor ET (later-onset ET). However, we hypothesize that education may be a protective factor of progression to dementia in premotor ET because ET pathology could be relatively mild in this phase of disease. Further, prevalent ET patients are expected to obtain less a protective benefit of education because ET pathology could be more severe in this group.

## 2 Methods

### 2.1 Study population

The data for this research were derived from the Neurological Disorders in Central Spain (NEDICES) study, a longitudinal, population-based survey of the prevalence, incidence, and determinants of major conditions of the older population. These included dementia, cerebrovascular disease, PD, and ET.^[24–36]^ Detailed accounts of the study population and sampling methods have been published.^[24–26]^ The survey area consisted of 3 communities: Margaritas (approximately 14,800 inhabitants), a working-class neighborhood in Getafe (Greater Madrid); Lista (approximately 150,000 inhabitants), a professional-class neighborhood in the Salamanca district (Central Madrid); and Arévalo (Ávila) (approximately 9000 inhabitants), an agricultural zone located 125 km northwest of Madrid. In each community, eligibility was restricted to residents who were aged 65 years or older and those who were present on December 31, 1993, or during 6 or more months of 1993. Eligible persons who had moved away from the survey area were not traced. In Margaritas and Arévalo, every eligible subject was selected for screening. However, because of the large number of older residents in Lista, proportionate stratified random sampling was used to select a subsample of 2113 subjects for screening. The selected study population was 6395 people, but 481 people were ineligible (census issues, address errors, or death), leaving 5914 eligible subjects, of whom 5278 were enrolled.

All procedures were approved by the ethical standards committees on human experimentation at the University Hospitals “12 de Octubre” (Madrid) and “La Princesa” (Madrid). Written (signed) informed consent was obtained from all enrollees.

### 2.2 Study evaluation

Face-to-face evaluations were performed at baseline (1994–1995) and then at follow-up (1997–1998). Briefly, at the time of their baseline assessment (1994–1995) and follow-up assessment (1997–1998), older subjects were interviewed face-to-face using a screening questionnaire to collect data on demographics, medications, current medical conditions, smoking (ever vs never), and drinker (ever/at least once per week vs never). The questionnaire included screening items for neurological disorders (dementia, cerebrovascular disease, PD, and ET). A short form of the questionnaire was mailed to subjects who refused or were unavailable for face-to-face interview. According to a recently published comorbidity score developed in ambulatory care settings,^[37]^ a comorbidity index was calculated. The presence of several conditions (atrial fibrillation, nonmetastatic cancer, metastatic cancer, chronic obstructive pulmonary disease, depression, dementia, diabetes, epilepsy [treated], heart failure, myocardial infarction, psychiatric disorders, renal disease, and stroke) resulted in the assignment of more points than others, and the score ranged from 0 to 28 (i.e., all conditions present).^[37]^

As in prior studies, at baseline, subjects were asked to rate their current health on a 5-point scale using the question, “In general terms, how would you describe your health: very good, good, fair, poor, or very poor?” A small number of subjects were in several categories (e.g., there were only 91 who described their health as very poor). Therefore, as suggested in several previous studies,^[38,39]^ we collapsed response options into 3 categories. These 3 categories were very good/good, fair, and poor/very poor.

One screening question for ET was included (“Have you ever suffered from tremor of the head, hands, or legs that has lasted longer than several days?”).^[34,35]^ This question was a Spanish adaptation of that used by the Italian Longitudinal Study on Aging (ILSA) Working Group.^[40]^ To assess the performance of this screening question, a random sample of approximately 4% of those who had screened negative was selected and contacted (n = 205). Of the 205 subjects who were contacted, 183 were successfully scheduled for an examination by a senior neurologist who routinely evaluates patients with movement disorders (JO; see http://www.ciberned.es/estudio-nedices) During the neurologic examination, participants were asked to perform 3 manual tasks to assess postural and kinetic tremors including sustained bilateral arm extension, bilateral finger-nose-finger maneuver (with a minimum of 6 repetitions with each arm), and an Archimedes spiral drawn with the dominant arm. The diagnostic criteria for ET were similar to those used in the Sicilian study (see below),^[41]^ and none (0%) of the 183 subjects was found to have ET, indicating that use of the screening question was likely to yield few false-negatives.^[35]^ The screening protocol for dementia included a 37-item version of the Mini-Mental State Examination (37-MMSE)^[42]^ and an 11-item version of the Pfeffer Functional Activities Questionnaire (FAQ).^[11]^ This screening protocol for dementia was designed and validated in a World Health Organization Aging Study, which included the investigation of interobserver agreement among international investigators with expertise in dementia.^[43]^ The sensitivities of both the 37-MMSE and the Pfeffer FAQ scale were greater than 90%.^[43]^

### 2.3 Neurological examination and neuropsychological test battery

At baseline and at follow-up, participants who screened positive for dementia or ET underwent a neurological examination at National Health Service clinics or at home. Participants were considered to have screened positive for dementia if: they scored <23 points on the 37-MMSE and >5 points on the Pfeffer FAQ scale; there were missing values (i.e., participant failed to provide an answer) on the 37-MMSE or Pfeffer FAQ scale; or the participant or proxy provided information of a history of cognitive decline.^[27,28]^ Participants were considered to have screened positive for ET if they answered “yes” to the 1 screening question for ET.^[34,35]^

The neurological examination was composed of a general neurological examination, a mental status examination, and the motor portion of the Unified Parkinson Disease Rating Scale (UPDRS).^[44]^ The details of the tremor examination have been presented previously.^[34,35]^ In addition, regardless of their screening results and diagnosis, participants underwent a short neuropsychological test battery.^[10,45]^ This battery has been described in other studies, and it consisted of the Trail Making Test-A, verbal fluency, recall (verbal and visual), and naming.^[46,47]^

### 2.4 Diagnosis of dementia and ET

The diagnosis of dementia was made by consensus of 2 expert neurologists. The medical records of all participants who received a diagnosis of dementia were also reviewed by a senior neurologist (FB-P) with the aid of a psychologist (FS-S, see http://www.ciberned.es/estudio-nedices). If there were doubts about any aspect of the dementia diagnosis, additional information (mainly from family doctors) was elicited. For the diagnosis of dementia, we applied the Diagnostic and Statistical Manual of Mental Disorders (DSM)-IV criteria.^[48]^ If dementia was diagnosed, data on age of onset were elicited. For this study, we categorized the different types of dementia into possible or probable AD, according to the National Institute of Neurological and Communicative Disorders and Stroke and the Alzheimer's Disease and Related Disorders Association criteria,^[49]^ and into other non-AD dementia subtypes.

Diagnostic criteria for all ET patients were similar to those used in the Sicilian study^[41]^ and have been presented previously.^[34,35]^ ET patients identified by 1 NEDICES neurologist were subsequently examined by 2 additional NEDICES neurologists. That is, the patients were classified as having ET only when the 3 neurologists agreed.

For participants who could not be examined (those who died before follow-up or those with screening, but who refused clinical examination), medical records were obtained from their general practitioners, from in-patient hospitalizations, and from neurological specialists (if they had visited one). In addition to these medical records, death certificate diagnoses were reviewed for each screened participant who had died before their neurological examination. Based on these sources of information, the diagnosis of dementia and ET was assigned using DSM-IV criteria, and those used in the Sicilian study, respectively.^[41,48]^

### 2.5 Final selection of participants

**Figure 1:**
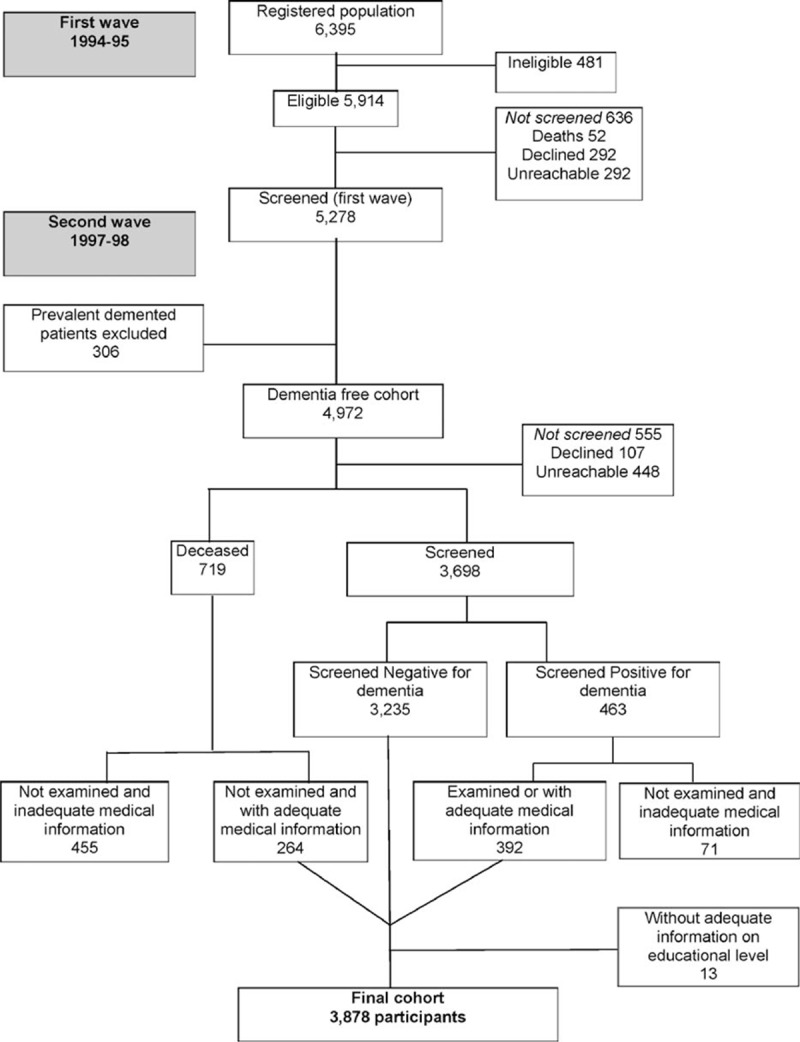
Flow chart of the study.

The flow chart at each step of the NEDICES survey is shown (Fig. 1). Of the 5278 participants screened for neurological disorders at baseline (1994–1995), we detected 306 prevalent dementia patients. These were excluded, leaving 4972 participants without baseline dementia. Of these, 555 were lost to the follow-up. Of the remaining participants, sufficient data were available on 3878 (78 premotor ET patients, 206 ET patients, and 3594 controls) who completed the follow-up evaluation, which consisted of a screening questionnaire and a neurological examination, and had information about educational level (Fig. 1).

### 2.6 Statistical analyses

All statistical analyses were performed using SPSS, version 21.0 (IBM Corp., NY). Age, number of medications, comorbidity index, and ET duration were not normally distributed (for each Kolmogorov–Smirnov test, *P* < 0.05), even after log transformation. Therefore, we used Mann–Whitney or Kruskal–Wallis tests to analyze these continuous variables, whereas the chi-square test was used to analyze categorical variables.

We used Cox proportional-hazards models to estimate hazard ratios (HRs) for dementia incidence with 95% confidence intervals (CIs). Participants were separated according to their educational attainment in lower education (illiterates or subjects who were capable of reading and writing) versus higher education (those with certificate of primary school or higher) participants. Patients and controls were then categorized into 6 groups: lower education premotor ET patients, higher education premotor ET patients, lower education prevalent ET patients, higher education prevalent ET patients, lower education controls, and higher education controls. This latter group was the reference category in the Cox proportional-hazards models.

In participants without incident dementia, a person-years variable was calculated using the time between the baseline and the follow-up evaluation or death in those who died before follow-up evaluation. By contrast, in participants who developed incident dementia, person-years were estimated using the time between the baseline evaluation and the reported date of dementia onset. When the date of onset of dementia was unknown, person-years were calculated as the midpoint between the first evaluation and the follow-up evaluation. In adjusted Cox proportional-hazards analyses, model 1 used a more restrictive approach considering all baseline variables that were associated (*P* < 0.05), both with the 6 groups of exposure (prevalent ET, premotor ET, and controls, according to high vs low education) and the outcome (dementia incidence). After that, we considered baseline variables that were associated (*P* < 0.05) with either the exposure or the outcome in bivariate analyses (i.e., a less restrictive approach) (model 2). Age (years), sex, smoker (ex-smoker plus current smoker vs never), consumption of ethanol (ever at least once per week vs less than one time per week), number of medications, comorbidity index, and self-rated health (good/very good, fair, and poor/very poor) were assessed at baseline and considered as potential covariates.

The presence of synergic interactions (synergy index S >1; S = OR [AB] − 1/[OR {Ab} − 1] + [OR {aB} − 1]) was based on the criteria described by Rothman (odds ratio [OR], Ab exposed to 1 factor, aB exposed to the other factor, AB exposed to both factors).^[50]^

## 3 Results

Among the participants, 3878 had a mean follow-up duration of 3.2 years (median 3.2 years, range 0.03–6.6 years). One participant without incident dementia died 1 month after the baseline evaluation (duration of follow-up 0.03 years), but the participant had adequate medical information to be included in the final sample of 3878 participants (Fig. 1). There were 206 participants with ET at baseline (204 diagnosed by direct examination and 2 by medical record review). Mean (median) ± standard deviation (SD) ET duration in the higher education prevalent ET patients was 12.9 (5.0) ± 15.1 versus 8.4 (6.0) ± 9.0 in the lower education ET patients (Mann–Whitney *U* test, *P* = 0.09) (Table 1). Of the 78 premotor ET patients who were included, 41 completed a face-to-face neurological examination at baseline and did not have ET. The 37 remaining premotor ET patients were re-interviewed during the follow-up evaluation to establish that the onset of their tremor had been after the baseline assessment. More important than self-reported information, however, was that baseline handwriting samples from these 37 premotor ET patients and 31 age-matched controls were blindly reviewed by one of the authors (EDL) and rated using Bain and Findley 10-point scale.^[51]^ None of the patients and controls had tremor that was in the ET range (all had Bain and Findley handwriting tremor scores ≤1, which are within the normal range).^[51]^

**Table 1:**
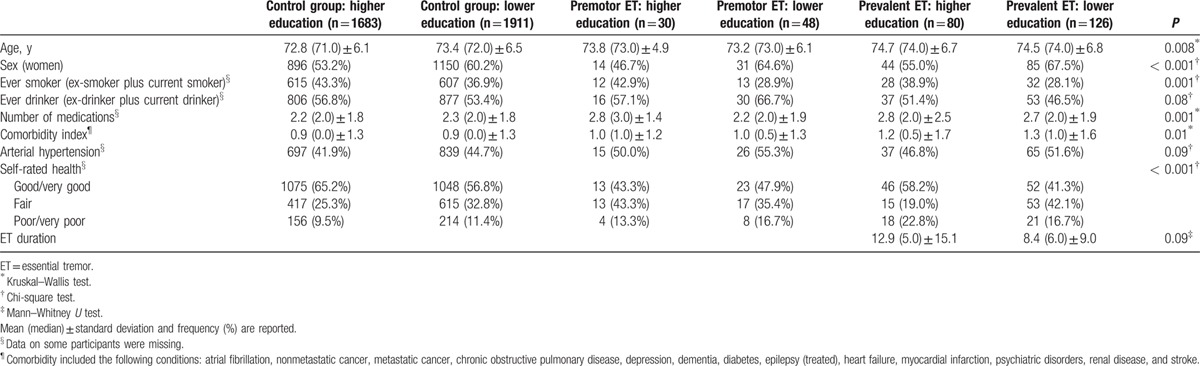
Baseline demographic and clinical characteristics of the 6 study groups.

Baseline characteristics of the patients and controls are shown in Tables 1 and 2. Among other differences, prevalent ET patients were older, had a higher comorbidity index, and took more medications than controls (Tables 1 and 2). In addition, prevalent ET and premotor ET patients reported their health as poorer or much poorer than controls (Tables 1 and 2), whereas the highest proportion of women was found in lower education prevalent ET patients (Table 1).

**Table 2:**
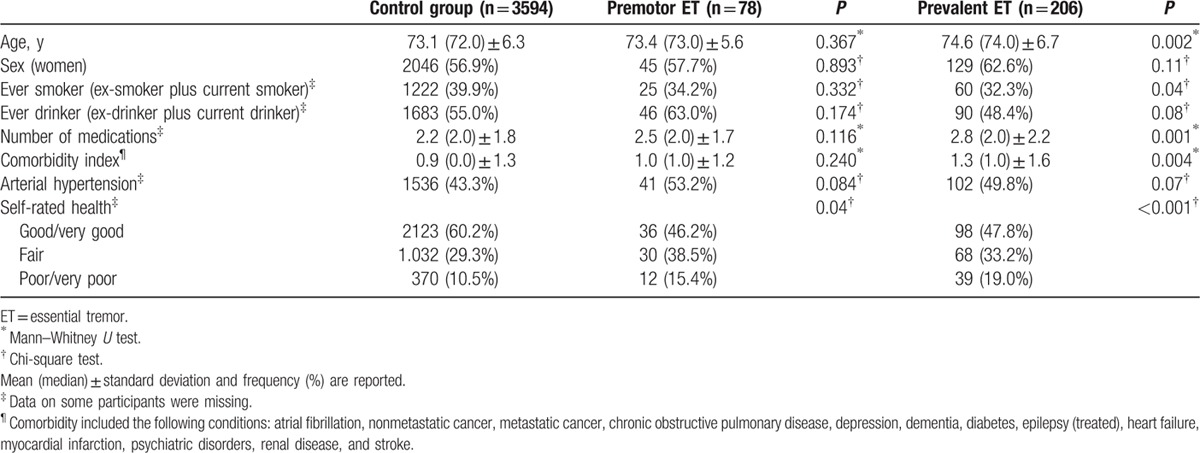
Baseline demographic and clinical characteristics of premotor ET patients versus controls and prevalent ET patients versus controls.

One-hundred sixty-one (4.1%) of 3878 participants developed incident dementia by the time of their follow-up evaluation. These 161 included 107 whose diagnosis was based on a follow-up examination and 54 who could not be examined but who had adequate medical information (medical records from general practitioners, in-patient hospitalizations, and neurological specialists, and also death certificate diagnoses). The mean duration of follow-up for these 161 participants was 1.7 years (median 1.5 years, range 0.5–6 years). Participants with incident dementia differed in several respects (baseline age, ever smoker, ever drinker, number of medications, and comorbidity index) from participants without incident dementia (Table 3). There was a trend towards a higher proportion of women among participants with incident dementia (Table 3). Further, they also reported their health as poorer or much poorer when compared with those without incident dementia (Table 3). The etiology of dementia was as follows: 115 (71.4%) AD, 18 (11.2%) vascular dementia, 11 (6.8%) dementia associated with parkinsonism (by definition none of these had ET), 6 (3.7%) secondary dementia, and 11 (6.8%) undetermined etiology. All 9 premotor ET patients who developed dementia were AD; meanwhile 11 (68.7%) out of 16 prevalent ET patients developed AD at follow-up. Nine (11.5%) of 78 premotor ET patients (unadjusted HR = 3.11, *P* = 0.001) and 16 (7.8%) of 206 prevalent ET patients (unadjusted HR = 2.17, *P* = 0.004) developed incident dementia versus 136 (3.8%) of 3594 controls (chi-square = 18.68, *P* < 0.001). The mean latency between the onset of ET and the onset of dementia in the 16 ET patients was 10.5 years (median 6.8 years, range 2.5–53 years). More specifically, 8 (16.7%) of 48 lower education premotor ET patients developed incident dementia versus 1 (3.3%) of 30 higher education premotor ET patients, 9 (7.1%) of 126 lower education prevalent ET patients, 7 (8.8%) of 80 higher education prevalent ET patients, and 92 (4.9%) of 1892 lower education controls (chi-square = 38.93, *P* < 0.001).

**Table 3:**
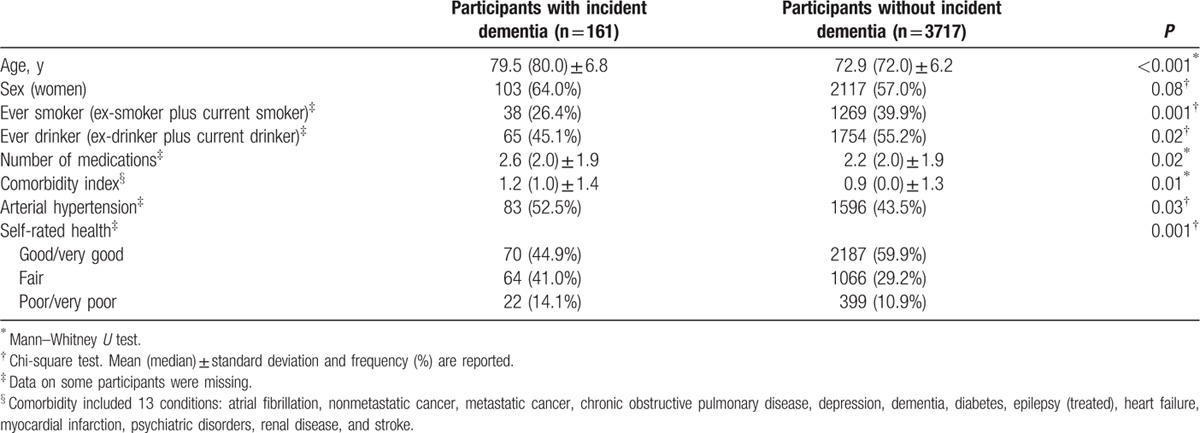
Baseline demographic and clinical characteristics of groups with and without incident dementia.

Table 4 summarizes the Cox regression models for all dementia subtypes and AD after adjusting for the effect of a range of covariates. As shown, ET patients (premotor and prevalent patients) had an increased risk of incident dementia. This effect was consistent in all models for premotor patients, whereas prevalent ET patients showed a significant trend when models were adjusted. The sample was stratified into 6 groups considering the diagnosis and education level (high vs low education). With the high education control group as the reference category, all groups, except higher education premotor ET patients, showed a significant increased risk of dementia (Table 4). In addition, a synergic interaction effect between status (disease or predisease and low education) was observed in premotor ET (S = 2.21), but not in prevalent ET (S = 0.93).

**Table 4:**
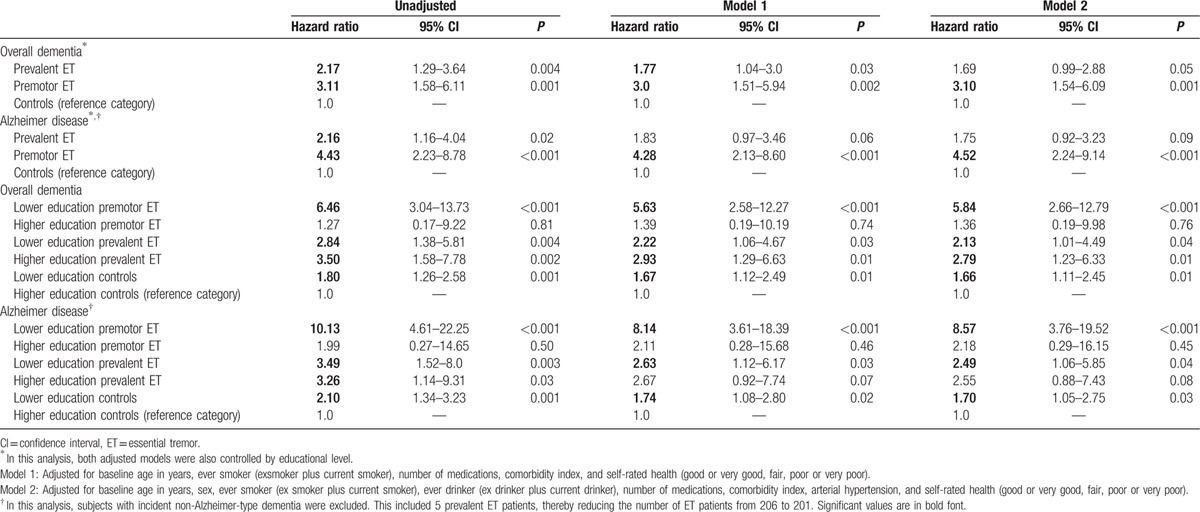
Risks of incident dementia and Alzheimer disease for the groups, according to the educational attainment.

### 3.1 Supplemental analyses

To compare a potential different effect of education on dementia between both control and ET groups, we conducted an additional Cox regression analysis in which the reference category was lower education controls. The HRs for incident dementia (model 1) were 13.35 (95% CI 1.61–6.97, *P* = 0.001) (lower education premotor ET patients); 1.32 (95% CI 0.66–2.65, *P* = 0.43) (lower education prevalent ET patients); and 0.59 (95% CI 0.40–0.88, *P* = 0.01) (higher education controls). In a fully adjusted Cox proportional-hazard model (model 2), the HRs for incident dementia were 3.50 (95% CI 1.67–7.32, *P* = 0.001) (lower education premotor ET patients); 1.33 (95% CI 0.66–2.67, *P* = 0.42) (lower education prevalent ET patients); and 0.59 (95% CI 0.40–0.88, *P* = 0.01) (higher education controls).

We also conducted a sensitivity analysis in which we excluded the 7 premotor and 5 prevalent patients who had neither an in-person examination at baseline nor an in-person examination at follow-up, and the 20 incident dementia patients who had neither an in-person examination at baseline nor an in-person examination at follow-up. In an adjusted Cox proportional-hazard model (model 1), the HRs for incident dementia were 7.80 (95% CI 3.51–17.35, *P* < 0.001) (lower education premotor ET patients); 1.97 (95% CI 0.27–14.52, *P* = 0.51) (higher education premotor ET patients); 2.86 (95% CI 1.33–6.15, *P* = 0.007) (lower education prevalent ET patients); 3.89 (95% CI, 1.68–8.99, *P* = 0.001) (higher education prevalent ET patients); and 1.97 (95% CI 1.27–3.08, *P* = 0.003) (lower education controls). In a fully adjusted Cox proportional-hazard model (model 2), the HRs for incident dementia were 8.35 (95% CI 3.73–18.67, *P* < 0.001) (lower education premotor ET patients); 1.91 (95% CI 0.26–14.13, *P* = 0.52) (higher education premotor ET patients); 2.90 (95% CI 1.35–6.24, *P* = 0.006) (lower education prevalent ET patients); 3.74 (95% CI 1.62–8.66, *P* = 0.002) (higher education prevalent ET patients); and 1.97 (95% CI 1.27–3.08, *P* = 0.003) (lower education controls).

## 4 Discussion

In this population-based prospective study, we present unique data about the effects of education on the risk of dementia in premotor and motor ET. Higher educational attainment may ameliorate the risk of incident dementia during the premotor phase of ET, but not during the motor phase of ET. The critical question is why education protects against dementia differentially in premotor versus motor patients. It may be that there is a critical point of neuropathological burden, in which the protective effect of education on dementia incidence is diminished or null. Moreover, it is intriguing that HRs for incident dementia in this cohort were higher in lower education premotor ET patients than in prevalent ET patients. The age of tremor onset in premotor ET patients is greater than that of prevalent ET patients, and risk of cognitive impairment has been more strongly linked with older onset patients,^[14–16,52]^ which could explain the increased HR we found in premotor ET patients.

The biological basis for the association of ET and cognitive impairment is not well-understood, but there are several hypotheses. First, ET is associated with abnormal brain connectivity involved in specific cognitive processes.^[53]^ In a recent study using resting-state functional magnetic resonance imaging,^[53]^ in at least 3 networks (default mode network and frontoparietal networks), increased connectivity was associated with worse performance in different cognitive domains (attention, executive function, visuospatial ability, verbal memory, visual memory, and language) and depressive symptoms. Second, some studies have demonstrated the presence of brainstem Lewy bodies in ET patients,^[23]^ which raises the question as to whether ET patients who develop dementia are more likely to have Lewy body pathology. Finally, although ET itself is not a tauopathy (i.e., a class of neurodegenerative disorders whose main features are accumulation of hyperphosphorylated tau protein), ET may predispose individuals to accumulate more widespread cellular tau aggregates, and thus tau could play a central role in the cognitive impairment that can accompany ET, as some evidence would indicate.^[54,55]^

Cognitive reserve posits that some individuals are able to cope more efficiently with manifestations of brain damage due to a more efficient utilization of brain networks.^[6]^ Epidemiological studies have shown that life experiences related to mental activities (e.g., education or occupation) are associated with decreased risk for incident dementia.^[2]^ In premotor ET patients, education may act against dementing processes through active reserve or compensatory capacity by facilitating recruitment of alternative brain networks. However, once a certain threshold is reached, and the amount of brain disconnection overshadows cognitive reserve mechanisms, the protective effect of education becomes less significant, as it is shown in ET patients with motor symptoms.

This study had limitations. First, it is possible that premotor and prevalent ET patients at baseline who subsequently developed dementia were really patients of AD with mild tremor rather than ET. However, this is unlikely because chronic action tremor is not an associated sign of AD.^[56]^ In addition, this was a study of incident dementia; hence, we excluded all subjects who were demented at baseline. Second, we did not explore the pathological or imaging correlates of education on brain networks. Structural and functional imaging studies are being used more frequently to explore the neural correlates of cognitive reserve,^[57]^ and these approaches should be considered in future prospective studies. Third, one possibility is that some of our ET patients were misdiagnosed and that they actually had PD rather than ET. However, the ET diagnoses were based on standardized clinical criteria used in previous studies,^[34,35]^ and the dementia diagnoses were assigned by the consensus of trained neurologists. In addition, a UPDRS motor examination was conducted at baseline and at follow-up to assess motor features of parkinsonism; none of our ET patients had tremor at rest or other features of parkinsonism on these examinations and none was taking medication for PD. The main strengths of this study include the standardized assessment and diagnostic criteria for ET, the detailed evaluation of incident dementia patients (i.e., uniform protocol applied by senior neurologists), and the prospective population-based design.
